# Evaluation of Health-Related Quality of Life among Tuberculosis Patients in Two Cities in Yemen

**DOI:** 10.1371/journal.pone.0156258

**Published:** 2016-06-03

**Authors:** Ammar Ali Saleh Jaber, Amer Hayat Khan, Syed Azhar Syed Sulaiman, Nafees Ahmad, Mohamed Saif Anaam

**Affiliations:** Department of Clinical Pharmacy, School of Pharmaceutical Sciences, USM, Penang, Malaysia; Indian Institute of Technology Delhi, INDIA

## Abstract

**Background:**

The health-related quality of life (HRQoL) of Tuberculosis (TB) patients is important because it directly influences the outcome of TB patients in several aspects. The current study aims to evaluate and to find the factors influencing the HRQoL of TB patients in two major TB-prevalent cities (Taiz and Alhodidah) in Yemen.

**Methods:**

A prospective study was conducted, and all TB patients meeting the HRQoL criteria were asked to complete the HRQoL SF-36 survey. The records of TB patients were examined for disease confirmation, and a follow-up was consequently performed for patients during treatment between March 2013 and February 2014 in Taiz and Alhodidah Cities. HRQol scores were calculated by using QM scoring software version 4.5, in which the physical component score (PCS) and mental component score (MCS) were obtained. The scores obtained between 47–53 normal based score (NBS) were considered equivalent to the US normal score. Low scores indicate the poor health situation of TB patients

**Results:**

A total of 243 TB patients enrolled in the study at the beginning of the treatment. A total of 235 and 197 TB patients completed the questionnaire at the end of the intensive phase (I.P.) and continuation phase (C.P.), respectively. The final dropout rate was 16.2%. The mean PCS and MCS scores at the beginning of treatment were low, thus showing the poor health situation of TB patients. The mean PCS scores at the beginning of treatment, end of I.P., and end of treatment were (36.1), (44.9), and (48), respectively. Moreover, the mean MCS score at the beginning of treatment, end of I.P., and end of treatment were (35.1), (42.2), and (44.3), respectively. The result shows that significant increases are observed at the end of I.P. for PCS and MCS because of the treatment and slight changes at the end of C.P. Despite this finding, the MCS score remains below the normal range (47), thus indicating a significant risk of depression among TB patients. Furthermore, general linear repeated measure ANOVA was performed for selected variables, to examine the changes of PCS and MCS over time. It was found that Alhodiah city, chewing khat habit, stigmatization, and duration of treatment more than six months were greatly associated with low mean MCS score of TB patient, indicating great risk of depression which may result in poor treatment outcome.

**Conclusion:**

TB patients in Yemen were found to have poor QoL, with a significant likelihood of depression. Highly risk depression was found among TB patients in Alhodiah city, khat chewers, stigmatization and having a duration of treatment more than 6 months. Therefore, additional efforts should be made to improve their QoL because it may affect the final clinical outcome of patients.

## Introduction

SF-36V2 health-related quality of life (HRQoL) is a generic self-reported questionnaire that is used to determine the quality of life (QoL) of the general population or individual patients [1]. Moreover, it is used to measure the influence and effects of the disease on patient functions and activities [2]. HRQoL acts as a predictor of patient therapy [3] by giving the health worker the characteristic changes of morbidity at a particular time and helping predict the factors that improve patient satisfaction [4] [5].

Several studies were conducted in different regions and countries to assess and evaluate the effect of tuberculosis (TB) on patients’ QoL [6], [7], [8],[9], [10]. Only a few studies have investigated TB at various treatment stages [11], [12], [9]. HRQoL evaluation is important because it predicts the change of health conditions of subjects at various levels of treatment and eventually affect the treatment outcome of TB patients [3], [4].

TB is considered a major disease, which impairs the daily life activities of the patient. The effect of TB on a patient’s health is considered essential [13] because, it can result to changes in the physical and mental states of the patient, and can consequently affect the treatment outcome [14]. Moreover, even less attention is given to QoL in developing countries as an influential factor, especially in TB disease [15].

WHO recognizes Yemen as an intermediate TB-burdened country [16]. TB in Yemen is considered one of the main health problems and ranks fourth in the priority list of public health issues [17]. In Yemen, the people’s standard QoL is considered low because of poverty, low life standard, and weak governmental health services [18], [19]. However, no study has performed HRQol for TB patients by using SF36 in the two major TB-prevalent cities in Yemen. This study aims to evaluate the HRQoL of TB patients in the two major TB-prevalent cities in Yemen for one year and to find the factors affecting the quality of life of TB patient during the treatment period.

## Study Population and Methods

This prospective study was conducted at the multicenter of the two major TB-prevalent cities in Yemen (Taiz and Alhodidah) under the National TB Control Program. Taiz City is located approximately 256 km south of Sana’a. Taiz holds the first rank regarding the number of population, with a population representing 12.16% of the total population of Yemen. Al-Hodeida City is located in the Red Sea Coast at a distance of 226 km from Sana’a; this city ranks second in terms of the number of population, with a population representing nearly 11% of the total population of the republic [17].

**Fig 1:**
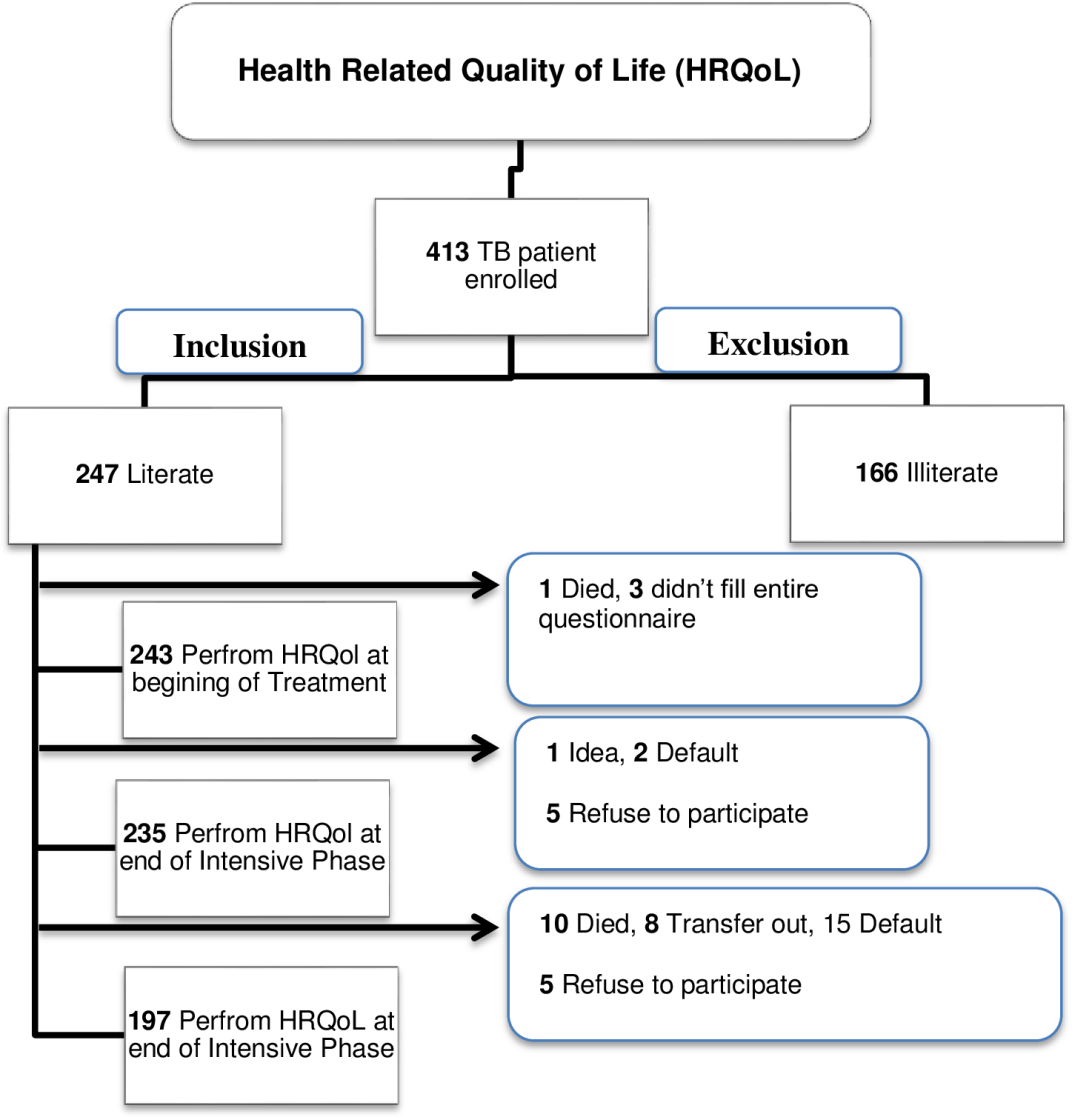
Number of TB patients enrolled in the study.

All the patients registered between March 2013 and February 2014, met the HRQoL criteria (18 years old and literate) [20], and confirmed their TB diagnosis upon enrollment in the study. Patients who defaulted, died, transferred out, and refused to participate were excluded from the study (Fig 1).

In Yemen, all TB patients who underwent diagnosed either in the private or governmental sector are advised to undergo treatments in the main TB centers under direct observed treatment (DOT).

TB patients should administrate the drug under DOT daily in intensive phase (I.P) and weekly in Continuous Phase. The treatment consists of an initial two month intensive phase of isoniazid (H), rifampicin (R), pyrazinamide (Z) and ethambutol (E), followed by a four month continuation phase of H and E [17]. The TB centers screen new TB patients on the basis of the guidelines of the Yemen National TB Control Program [17]. Given that the studies were conducted at the multicenter of the two major TB-prevalent cities in Yemen, the data were collected by six trained pharmacists and nurse data collectors (three in each city). The patients who agreed to participate in the study were asked to sign an informed consent form after disclosing the clear purpose of the study. Certificates and permissions to use the SF-36V2 in Yemen were provided by Quality Metric Incorporated (USA).

The data questionnaire consists of two parts. The first part explored the socio-demographical information of TB patients, including the stigma and knowledge questionnaire. Moreover, a separate questionnaire was used to collect clinical characteristics from patients’ records, such as cough history and type, x-ray report, comorbidity, and a number of clinical signs at the beginning of the treatment. The second part of the questionnaire included an Arabic HRQol version and an official translation made by Quality Metric Incorporated. The forward translations of questionnaires were performed in Sana’a University by a group of Arabic native speakers. Backward translations were performed by the UN Organization office in Sana’a for the purpose of conceptual equivalence. HRQols were validated, and the Cronbach’s alpha obtained was 0.70. The HRQoL questionnaire consists mainly of eight domains that reflect various aspects of the QoL of TB patients, including physical functioning, Vitality, physical role, bodily pain, general health, social functioning, emotional role, and mental health.

### Statistical analysis

Data analyses were performed using PASW (Predictive Analysis Software, version 22, IBM Corp, Armonk, NY). The physical component score (PCS) and mental component score (MCS) were calculated using QM scoring software version 4.5 [21]. MCS and PCS were used as continuous variables for mean and standard deviation calculations. Furthermore, the other categorical variables were represented as percentage and counts. The scores of both health domains were considered normal if they are between 47 to 53. Scores below 47 were considered a sign of defective function [20], [7]. Moreover, a score of <43 in MCS indicates a significant risk of depression. Minimal clinical differences were considered if a variation of three points or less exists. Moreover, General linear model repeated measure ANOVA was performed to examine the changes of PCS and MCS over time at three stages of treatment, and to find whether independent variables plays a role in changing PCS and MCS score over time. Effect size was consider to be small (partial eta squared = 0.01), moderate (partial eta squared = 0.06) and large (partial eta squared = 0.14) [22]. Independent variables consider being significant if p-value is < 0.05. In addition, Independent sample t-test is used to compare the mean PCS and MCS score of a khat chewer and non-chewer, in addition to smoking and nonsmoking TB patient over time.

### Ethics

The studies were approved by the National Committee for Health and Medical Research and were registered under the Ministry of Public Health and Population in Sana’a-Republic of Yemen. Furthermore, the studies were approved by the National TB Control Program of Yemen.

## Result

### Socio-demographic characteristics of Tuberculosis Patients

During the study period of data collection, a total of 628 new TB patients were register in Taiz and Alhodidah city. Among them, 413 TB patients agreed to participate in the study. Moreover, by applying the HRQol criteria (18 years old and literate) [20], only 247 patients were eligible for the study. Among the 247, 243 agreed to participate at the beginning of treatment, with a response rate of 93%. During intensive phase (I.P.) treatment, 1 patient died, and 3 refused to complete the survey. A total of 235 participants enrolled at the end of I.P., with a dropout rate of 3.3%. During the C.P. of treatment, 10 (4.3%) patients died, 8 (3.4%) transferred out, 15 (6.4%) defaulted, and 5 (2.1%) refused to participate, demonstrating a dropout rate of 16.2%. Approximately, 38 patients were excluded from the survey during C.P., demonstrating a dropout rate of 19.5%. Only 197 TB patients were enrolled at the end of the treatment.

The mean age of TB patients was 27.4 (SD 10) years old and ranged from 18 years old to 84 years old. The male–female ratio was 1.96:1. The majority of patients were males (66.3%), smear positive (62.6%), living in urban areas (69.1%), had habits of chewing khat (62.6%), nonsmokers (72%), had an average age of ≤45 years old (93.8%), and had no family history of TB (69.5%). Over half of the patients were from Taiz City (59%), not married (53.9%), and had a monthly income below 10,000 Rial (55.6%), B.M.I with ≥ 18.5 kg /m2 (55.6%). Regarding educational status, the highest and lowest proportion of patients obtained primary education (37%) and university education/university certification (9.5%), respectively (Table 1).

**Table 1:** Tuberculosis Patient Characteristics (for 243 TB patients)-In the beginning of the treatment for both cities.

Tuberculosis features	Patient n (%)
**Gender**	
Male	161 (66.3)
Female	82 (33.7)
	**243(100)**
**Form of TB**	
Smear Positive TB	152 (62.6)
EPTB ^**¶**^	76 (31.3)
Smear Negative TB	15 (6.2)
	**243(100)**
**Age**	
>45	15 (6.2)
≤45	228 (93.8)
	**243(100)**
**Governance**	
Taiz	144 (59.3)
Alhodiah	99 (40.7)
	**243(100)**
**Area** ^**€**^	
Urban	168 (69.1)
Rural	75 (30.9)
	**243(100)**
**Marital status**	
Married	112 (46.1)
Non-married	131 (53.9)
	**243(100)**
**Occupation**	
Employed	125 (51.4)
Non Employed	118 (48.6)
	**243(100)**
**Income (Rial) °**	
≤10,000 or don’t have	135 (55.6)
>10,000	108 (44.4)
	**243(100)**
**Smoking status**	
Smoker	68 (28)
Non smoker	175 (72)
	**243(100)**
**Chewing khat** ^**ǂ**^	
Yes	152 (62.6)
No	91 (37.4)
	**243(100)**
**Stigma**	
Yes	99 (40.7)
No	144 (59.3)
	**243(100)**
**Knowledge**	
Poor	21 (8.6)
Good	222 (91.4)
	**243(100)**
**Family history of TB**	
Yes	74 (30.5)
No	169 (69.5)
	**243(100)**
**BMI** ^**z**^	
< 18.5 kg/m2	108 (44.4)
‎≥‎ 18.5 kg/m2	135 (55.6)
**BCG**	
Yes	63 (25.9)
No	180 (74.1)
	**243(100)**
**History contacts with TB Patient**	
Yes	31 (12.8)
No	212 (87.2)
	**243(100)**
**Primary Education**	
Yes	90 (37) 1
No	153 (63)
	**243(100)**
**P.Secondary Education ‴**	
Yes	59 (24.3) 3
No	184 (75.7)
	**243(100)**
**Secondary Education**	
Yes	71 (29.2) 2
No	172 (70.8)
	**243(100)**
**University**	
Yes	23 (9.5) 4
No	220 (90.5)
	**243(100)**

^**¶**^ EPTB: Extra Pulmonary Tuberculosis

^**€**^ Area: Governance

**°** Rial: Yemen’s currency

^**ǂ**^ khat: shrub that grows in parts of East Africa and Yemen

^**z-**^BMI: Body Mass Index

^**>**^ I.P: Intensive Phase

^**<**^ C.P: continuous Phase

‴ P.Secondary: preparatory secondary.

Clinical characteristics of TB patients at the beginning of treatment show that majority of patients suffer from cough history lasting for over three weeks (68.7%). Furthermore, approximately 67.5% of patients underwent x-ray examinations. Moreover, the majority of TB patients had over four clinical symptoms at the beginning of treatment (88%). Over half of the patients had a productive cough (58.4%), and only 16.5% of patients were found to be comorbid (Table 2).

**Table 2:** Clinical characteristics for TB patient-Beginning of treatment for both cities.

Tuberculosis features	Patient n (%)
**Cough History**	
>3 weeks	167 (68.7)
< 3 weeks	50 (20.6)
No cough	26 (10.7)
	**243(100)**
**Cough Type**	
Productive	142 (58.4)
Non- productive	79 (32.5)
No cough	22 (9.1)
	**243(100)**
**X-Ray**	
Performed	164 (67.5)
Not performed	79 (32.5)
**X-Ray lesion**	
Bilateral	56 (23)
Unilateral	100 (41.2)
No lesion	9 (3.2)
No test performed	78 (32.1)
	**243(100)**
**Lungs cavities**	
Single cavity	64 (26.3)
Two cavity	43 (17.7)
Multi-cavity	15 (6.2)
No cavity	42 (17.3)
No test performed	79 (32.5)
	**243(100)**
**Comorbidity**	
Yes	40 (16.5)
No	203 (83.5)
	**243(100)**
**Clinical symptoms beginning of treatment**	
> 4	214 (88.1)
≤ 4	29 (11.9)
	**243(100)**

To implement this study, an Arabic SF 36 version questionnaire was developed. An internal consistency test was performed by using Cronbach's alpha. Cronbach’s alpha was found to be 0.70 (Table 3).

**Table 3:** Item Internal consistency of Arabic Version of HRQoL SF-36V2.

S.NO	Scale	Internal consistency (Cronbach’s Alpha)-Arabic version
**1**	HRQoLV2	0.707

HRQoL was measured using the normal based score (NBS). NBS scores ranging from 47 to 53 were considered equivalent to US population norms [20]. All of the 8 domains at the beginning of the treatment were found to obtain an NBS of less than 47. At the end of the I.P., most of the domain scores were less than 47, except GH and VT, which had scores of 50.5 and 47.5, respectively. Moreover, a change of 3 NBS or more was considered the minimal clinically significant difference. A change of more than 3 NBS found at the end of I.P. was compared with the baseline in all 8 domains. At the end of the treatment, a change of 3 points for PF, BP, GH, and VT was observed. Moreover, only GH and VT obtained scores of more than 47 at the end of I.P. At the end of the treatment, the GH, VT, and PF scores were more than 47 NBS (Table 4).

**Table 4:** Health Related Quality of Life SF 36V2 scores using Norm Based Scoring (NBS) at various stage of treatment.

Scale	Mean score (SD)		
	Beginning of I.P (N = 243)	End of I.P (N = 235)	End of C.P (N = 197)
PF*	28.9 (10.7)	41.3 (8.5)	47.6 (9.9)
**RP** ^**ǂ**^	31.2 (7.7)	40.8 (9.2)	43.9 (11)
**BP**‴	33.5 (9.3)	42.6 (8.6)	46.2 (10.5)
**GH**^**¶**^	44.7 (11.7)	50.5 (10.6)	52 (11.2)
**VT** ^**ˠ**^	40.5 (10.2)	47.5 (10.4)	51.1 (12.4)
**SF**^z^	39.1 (9.9)	42.7 (8.4)	43.4 (8.6)
**RE**^**ʏ**^	27.1 (9.3)	38.5 (11.2)	42.4 (13.5)
**MH**^**€**^	32.4 (11.7)	41.7 (13.3)	44.7 (14.8)

* PF: Physical Functioning

^**ǂ**^ RP: Role-Physical

‴ BP: Bodily-Pain; GH

^**¶**^ General-Health

^**ˠ**^ VT: Vitality

^z^ SF: Social-Functioning

^**ʏ**^ RE: Role-emotion

^**€**^ MH: Mental-Health

The 8 domains are compiled to 2 overall measures of health, namely, MCS and PCS. PCS and MCS average scores at the beginning of treatment were 36.1 (SD = 6.6) and 35.1 (SD = 11.3), respectively, which reflect the worst mentality and physical activity due to TB disease in all forms of TB. Significant changes were observed at the end of I.P., with a difference of 7.8 and 6.1 NBS for PCS and MCS, respectively. At the end of the treatment, only the PCS mean score was found to be over 47, which reflects the significant improvement in physical activity with the treatment compared with MCS, indicating a low score of 44.3. At the end of I.P., MCS indicated that TB patients were under the risk of depression; the score of MCS slightly increased during C.P., and shows that TB patient crosses the likelihood of depression when compared at the end of I.P. (Table 5).

**Table 5:** Changes in Physical and Mental Component Summary (PCS and MCS) during various stages of treatment.

Component Summary	Mean score (SD)			Alteration of Mean Score
	Start of I.P (N = 243)	End of I.P (N = 235)	End of C.P (N = 197)	
PCS*	36.1 (6.6)	44.9 (6.6)	48.9 (7.8)	12.8
**MCS**^**ǂ**^	35.1 (11.3)	42.2 (11.7)	44.3 (13.1)	9.2

* **PCS:** Physical component score

^**ǂ**^ **MCS**^:^ Mental Component Score

To find the changes of HRQol for selective variables at 3 stages of treatment, PCS and MCS mean score was analyzed. Table 6 Shows that PCS mean score for the both governance, chewing khat habit, stigma, and duration of treatment improved during the three time point of TB treatment and cross 47 NBS at the end of treatment. On the other hand, with respect to governance, the MCS mean score at the end of treatment for Alhodiah city was low (39.2) compare to Taiz city (47.4). Likewise, MCS mean score in the end of treatment for chewing khat and having stigmatization was 39.8 and 41.9 respectively shows low score.

**Table 6:** Changes in Physical and Mental Component Summary (PCS and MCS) among selected variables.

Variable	PCS* Mean score (SD)			MCS^ǂ^ Mean score (SD)		
	Start of I.P (N = 243)	End of I.P (N = 235)	End of C.P (N = 197)	Start of I.P (N = 243)	End of I.P (N = 235)	End of C.P (N = 197)
**Governance**						
Taiz	34.4±6.2	44.5±6.4	49.3±7.6	39.8±7.9	45.7±9.8	47.4±11.1
Alhodiah	38.6±6.5	45.6±6.7	48.3±7.6	28.5±12.4	37.3±12.5	39.2±14.5
**Chewing khat**						
Yes	34.9±6.2	44.9±6	48.2±8.4	30.4±12.9	38.2±13.3	39.8±15.8
No	37.7±6.9	45.1±7.3	49.3±7.5	38.2±8.7	44.6±9.7	47±10.3
**Stigma**						
Stigmatization	35.9±6.5	44.3±6.7	48.6±8.3	32±11.4	38.9±12.8	41.9±14.7
Non Stigmatization	36.1±6.8	45.3±6.5	49.1±7.5	37.3±10.9	44.4±10.3	46±11.6
**Treatment duration**						
6 months	36.2±6.5	45.3±6.6	49.7±8.1	37.1±10.3	43.3±10.8	45.1±12.5
>6 months	36.1±6.4	44.3±6.3	47.7±7.3	32.8±11.8	40.6±12.4	43.3±13.7
**Smoking status**						
Yes	33.6±5.4	44±5.8	48.7±7.8	34.7±11.4	41.6±11.8	43.6±13.1
No	37.2±6.8	45.4±6.9	48.9±7.9	36.2±11.5	44±11.4	45.9±13

* **PCS:** Physical component score

^**ǂ**^ **MCS**^:^ Mental Component Score

Repeated measure anova (Table 7) shows that Governance (df = 1.745, F = 16.489), Smoking (df = 1.705, F **=** 5.632), chewing khat (df = 1.723,F = 10.381), ≥ 3 TB symptoms at start of treatment (df = 2, F = 4.641) interact with time to predict trend in PCS score. In addition, Table 8 shows that, Governance (df = 1, F = 48.164), chewing khat (df = 1, F = 26.685), stigma (df = 1, F = 11.710), Total treatment duration (df = 1, F = 4.002) were the predictors of difference of MCS score. This is reflecting by the difference between groups in 3 times stages of treatment.

**Table 7:** General Linear Model Repeated measure ANOVA test for physical and mental component score within the subject effect.

**SOURCE**	**df**	**F**	**P-Value**	**Partial eta Squared**
**Physical Component Score**			
Time*Gender	1.688	1.189	0.300	0.006
Time*Governance	1.745	16.489	**0.001**	0.078
Time*Marital status	1.688	0.433	0.615	0.002
Time*Employment	1.692	1.288	0.274	0.007
Time*Income (Rial)°	1.697	2.794	0.072	0.014
Time*Smoking	1.705	5.632	**0.004**	0.028
Time*chewing khat ^ǂ^	1.723	10.381	**0.001**	0.051
Time*Stigma	1.686	0.234	0.753	0.001
Time*Knowledge	1.686	1.853	0.165	0.009
Time*Comorbidity	1.693	1.947	0.151	0.010
Time*≥ 4 TB symptoms at start of treatment	2	4.641	**0.010**	0.023
Time*Total Treatment Duration	1.693	1.886	0.160	0.010
	**Mental Component Score**			
Time*Gender	1.690	1.132	0.317	0.006
Time*Governance	1.687	1.954	0.151	0.010
Time*Marital status	1.685	0.326	0.685	0.002
Time*Employment	1.687	0.913	0.388	0.005
Time*Income (Rial) °	1.687	0.175	0.803	0.001
Time*Smoking status	1.687	0.016	0.972	0.000
Time*Chewing khat ^ǂ^	1.684	0.379	0.649	0.002
Time*Stigma	1.686	0.554	0.713	0.003
Time*Knowledge	1.686	0.287	0.713	0.001
Time*Comorbidity	1.687	0.191	0.789	0.001
Time*≥ 4 TB symptoms at start of treatment	2	0.452	0.637	0.002
Time* Total Treatment Duration	1.691	1.199	0.298	0.006

**°** Rial: Yemen’s currency

^**ǂ**^ khat: shrub that grows in parts of East Africa and Yemen

* Greenhouse-Geisser values as sphericity cannot be assumed (p < 0.0005).

**Table 8:** General Linear Model Repeated measure ANOVA test for the physical and Mental score in between the subject effect.

**SOURCE**	**df**	**F**	**P-value**		**Partial eta squared**
**Physical component score**				
Time*Gender	1	0.202	0.654		0.001
Time*Governance	1	3.175	0.076		0.016
Time*Marital status	1	0.388	0.534		0.002
Time*Employment	1	1.513	0.220		0.0081
Time*Income (Rial)**°**	1	1.594	0.208		0.008
Time*Smoking status	1	3.188	0.076		0.016
Time*Chewing khat ^**ǂ**^	1	1.204	0.274		0.006
Time*Stigma	1	0.966	0.327		0.005
Time*Knowledge	1	1.980	0.161		0.010
Time*Comorbidity	1	0.592	0.443		0.003
Time*≥ 4 TB symptoms at start of treatment	1	3.285	0.071		0.017
Time* Total Treatment Duration	1	1.534	0.217		0.008
	**Mental Component Score**				
Time*Gender	1	0.588	0.444	0.003	
Time*Governance	1	48.164	**0.001**	0.198	
Time*Marital status	1	0.073	0.787	0.001	
Time*Employment	1	0.731	0.394	0.004	
Time*Income (Rial) **°**	1	0.595	0.442	0.003	
Time*Smoking status	1	1.931	0.166	0.010	
Time*chewing khat ^**ǂ**^	1	26.685	**0.001**	0.120	
Time*Stigma	1	11.710	**0.001**	0.057	
Time*Knowledge	1	0.001	0.984	0.001	
Time*Comorbidity	1	3.166	0.077	0.016	
Time*≥ 4 TB symptoms at start of treatment	1	0.200	0.655	0.001	
Time* Total Treatment Duration	1	4.002	**0.047**	0.020	

**°** Rial: Yemen’s currency

^**ǂ**^ khat: shrub that grows in parts of East Africa and Yemen

* Greenhouse-Geisser values as sphericity cannot be assumed (p < 0.0005).

## Discussion

Few studies have been conducted to evaluate the TB status in Yemen. Our study focuses on two major TB-prevalent cities in Yemen with different climates and location. Moreover, all forms of TB were included in the study in the hope that the final finding of HRQol may play a role in improving the overall QoL of patients.

The total sample size of the study was 413. Among the patients, only 243 (58.8%) were found to be literate. The result shows that a high percentage of TB patients are illiterate in Yemen [17]. Another similar study was conducted in Yemen to determine the role of gender and literacy in the diagnosis and reported that a high percentage of TB patients are illiterate [23]. Contrary to our study, a survey was conducted to find the factors that affect the compliance of TB patients to treatment in Yemen reported; the survey reported that the majority of patients were literate [24]. Our finding is considered normal, considering that the majority of people in Yemen are illiterate.

Our study shows that the majority of TB patients were below 45 years old, demonstrating that those productive ages are under a major risk of contracting TB in Yemen. A similar prospective study was conducted in Yemen to identify the risk factor associated with non-compliance to TB treatment and found that the majority of TB patients enrolled in the study were of productive age [24]. Another study was conducted in North India to assess the HRQoL Pulmonary TB and reported that the majority of patients were in their productive age [25]. Contrary to this study, a survey was conducted in the United Kingdom to explore the health status of active TB patients and found that the majority of patients were old [11]. This result was due to the high percentage of elderly people in the United Kingdom.

A dropout rate of 19.5% was observed at the end of treatment, with an enrollment of only 197 TB patients. Despite this finding, the sample size was considered high. A similar study was performed in Penang, Malaysia, and found that the effect of HRQol on TB patients had a dropout rate of 29.2%, with only 153 PTB patients enrolled at the end of treatment [7]. The reason for the high dropout rate in our study is due to default.

Our finding shows that, the majority of patients were basically from urban areas (69%) because our study was conducted in two of the major prevalence cities in Yemen. Despite this result, 31% came from the rural area, thus concluding that TB patients prefer TB centers in cities because of several reasons: shortage of TB services in rural areas and non-availability of diagnosis facilities [26] [27]. This result reflects the tremendous shortage of TB services in rural areas. A similar study was performed in Pakistan to explore the factors that affect TB patients in rural areas and found that the shortage of TB services in rural areas forced patients to seek treatment in urban areas [28]. Other studies argued that this preference may be caused by the patients’ mistrust of the TB control programs in rural areas [29].

Although our study was conducted in two big cities, the majority of TB patients (55%) were found to be under the poverty line. Our finding reflects the hard financial situation of patients, where the majority of the Yemen population are under the poverty threshold, with daily income less than 1.25 $ [30]. Moreover, WHO states that Yemen is considered a low/middle-income country with a high percentage of poverty [31]. Furthermore, poverty in Yemen plays a significant role in increasing the burden of TB [17].

Our study shows that over 60% of TB patients are khat chewers. Chewing khat is considered a serious issue in Yemen because it results in severe consequences for people’s health. A supportive study conducted in Yemen reports that a majority of people are khat chewers. In Yemen, a survey conducted in 2015 explored the clinical and cytological effects of smoking and non-smoking khat chewers in Yemen and reported that the majority of people are chewing khat [19] [32]. Another Study conducted to find histopathological changes in oral mucosa due to khat shows that the main reasons for people chewing khat include the desire for psycho-stimulation effect, such as excitement. Feeling of excitement occurs because of the presence of cotinine [32].

The validity of HRQol was assessed, and our finding indicates that the SF-36 questionnaire has a Cronbach’s alpha of 0.70. Other studies reported a minimum Cronbach’s alpha of 0.70 [33], [7], [34], [9], [35], [36], [37], [38], [39], [40]. This finding shows that the SF-36 questionnaire can be applicable in Yemen.

The HRQoL obtained from TB patients differs from one study to another depending on the location and culture of the study [41]; furthermore, this was clearly observed in the significant variation of the results obtained from different studies. Our study finds that the HRQoL for all eight domains was low at the beginning of treatment, whereas others indicate low scores only in a few domains [2]. Little improvement in the score was found at the end of the treatment compared with those obtained at the end of the I.P. A similar study was conducted in Hamdan, western Iran, to assess the QoL of TB patients at the beginning of treatment, including end of I.P. and end of the treatment, and reported little improvements in the score at the end of the treatment [8]. A similar study was conducted in China to assess the QoL of TB patients and reported little improvement in the HRQol score at the end of C.P. compared with I.P. because of the treatment [9].

The study also found that at the beginning of the treatment, the PF and RE scores were the lowest domains scores, with 28.9 and 27.1 scores, respectively. A similar study was conducted in the United Kingdom to assess the HRQoL and found that RE was the most affected score compared with others at the beginning of the research [12], [42]. This finding reflects the worst severity emotional status of TB patients associated with TB disease. An extremely low score of RE may be due to the feeling of depression at the time of TB diagnosis [42]. Physical functioning is defined as the ability of the patient to perform basic daily life activity [43]. TB results in the restriction of daily activities due to the major signs and symptoms associated with the disease [37]. Our study indicates the low score of PF at the beginning of treatment; other studies report the same finding [12] [8] [44] [6] [42] [9]. However, With continuing treatment, a significant improvement of PF and RE occurring at the end of I.P. was observed, with scores of 41.3 and 38.5, respectively. A study was conducted to find HRQoL among latent and active TB adult and reported an enhancement of RE at the end of treatment course for active TB patients [42].A similar study was conducted in Canada to explore the health state of latent and active TB and reported an improvement of RE at the end of treatment [43]. Contrary to our finding, a study conducted in China states the non-improvement of RE-score at the end of I.P. [9].

Moreover, at the end of treatment, RE noted a slight improvement compared with that at the end of I.P. with a score of 42.4 only. This finding shows the TB patients are still under the risk of depression. Alternately, PF shows considerable improvement from 28.9 to 47.6 at the beginning of treatment and end of treatment, respectively, indicating significant improvement and quick response in physical compared with mental functioning due to treatment [43]. TB treatment received a positive effect on improving the QoL of TB patients. A systemic review was conducted to measure HRQoL in TB patients and reported the strong relation and a positive effect of TB treatment on improving the QoL of TB patients [14]. Despite this finding, the improvement in the normal range was found only in a few scores at the end of I.P. Moreover, GH and VT scores were 50.5 and 47.5, respectively, at the end of I.P. In Canada, a longitudinal cohort study was conducted to compare HRQol among patients treated with TB, and processed Latent TB, including the person screened but not treated with TB drugs, for a follow-up period of one year. The result shows a significant increase in GH and VT at the end of I.P. with treatment [42]. With continuing treatment, the score reaches to 52 and 51.1 for GH and VT, respectively. A similar study conducted in Malaysia reports an increase in VT score over 47 at the end of C.P. [7]. [6] states that the education level probably plays a role in enhancing vitality because education results in additional flexibility in life and motivates self-care, which leads to a decrease in physical problems and improve vitality, thereby improving physical functioning. Others state that an improvement of vitality may be due to the filling of energy brought about by the TB treatment [9], [12], [42], [45]. We conclude that TB treatment obtained a positive effect on improving the QoL of TB patients.

Scores less than 47 NBS for PCS and MCS were obtained at the beginning of the treatment. Similar to our finding, [11] finds compromised HRQoL among TB patients. Other studies show the low score of PCS at the start of I.P. compared with MCS [12]. Another study finds good QoL at the beginning of treatment [46]. Our study finds that with continuing treatment, the score had increased clearly at the end of I.P., providing a difference of 8.7 and 7.1 NBS for PCS and MCS, respectively, which reflects the significant changes in the mental and physical health of TB patients at this duration of time. Despite the occurring changes, scores were still below 47. At the end of the treatment, only PCS shows significant improvement to over 47 for all TB patients, thus suggesting that TB treatment improves the physical conditions of TB patients. A study conducted in the United Kingdom indicates that PCS was worse than MCS [42]. Another study finds that physical health improved and recovered extremely fast compared with mental health [14], [47]. Other studies reported a significant increase in MCS during the treatment phases [42], [43]. Others still find a substantial increase in MCS at the end of treatment compared with PCS [12]. The low score of MCS may result in increased default rate of TB patients at the end of the treatment [48]. The MCS score in our finding shows only 35 NBS, thus indicating the poor mental condition of TB patients. A similar finding was obtained by [12], which concludes that the beginning of treatment shows 36 NBS PCS. Others report a high score of 61 MCS and PCS [35]. Our study reports a slight improvement of score at the end of the 6^th^ month, other studies report the same [9], [10], [49]. Another study reports that no significant improvements existed in the health domain score during the overall treatment period among TB patient [50].

**Fig 2:**
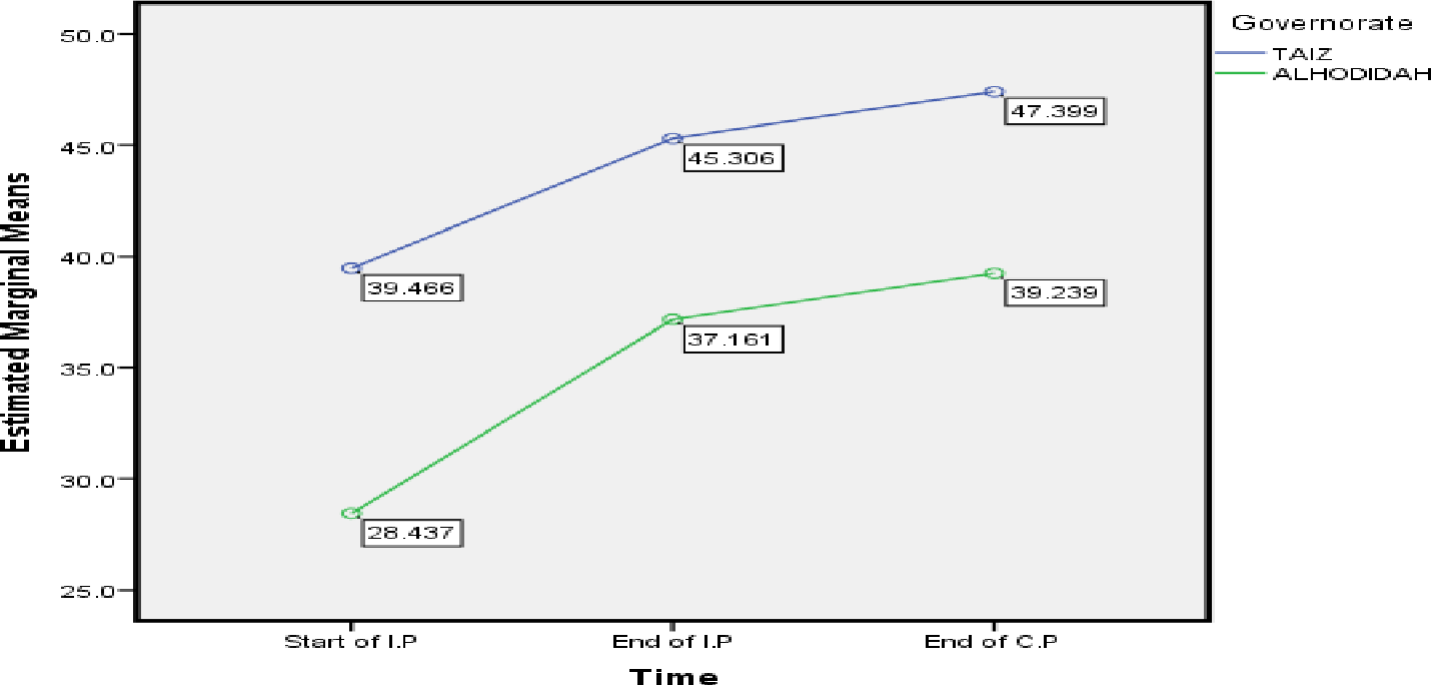
Taiz and Alhodidah Governance: Difference in estimated marginal mean of MCS.

Our study found that patient in Alhodidah governance, chewing khat, stigmatization and duration of treatment more than 6 months were the predictors of difference in mean MCS score among TB patients. With respect to governance, MCS mean score among TB patient in Alhodiah city was low in the three stages of treatment showing a risk of depression among TB patients (Table 6). In addition, Fig 2 illustrate the difference in MCS mean score between two the governance, Taiz city shows high MCS score comapre to Alhodidah in three different time’s points. A high score may be due to the higher educational level of people in Taiz city compare to Alhodidah. A similar study found that there is a great relation between MCS score and TB patient educational level [2] [51] [52]. Better educational level can be associated with more self-confidence, and positive attitude toward sickness and social states [53]. Others found the higher educational level the higher is the quality of life [53], [54] [14]. Therefore, educational status of TB patient can play an important role in affecing the MCS score which may affect the outcome of TB patient. Contrary to our study, [11] found that education level of TB patient is one of the main predictors of PCS score not MCS. In addition, [6], [55] report a great relation between education and PCS score.

**Fig 3:**
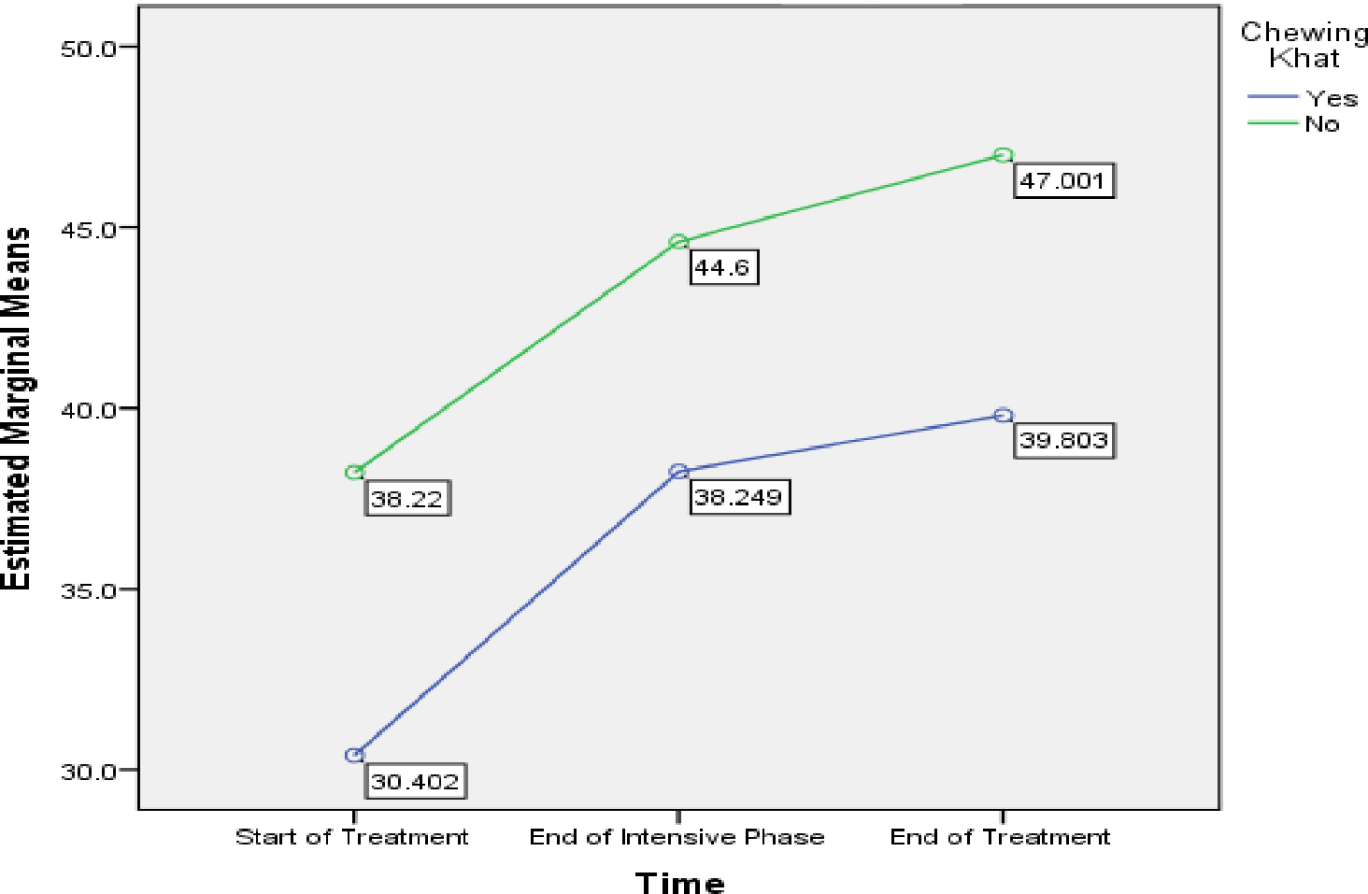
Chewing Khat Stuatus: Difference in estimated marginal means of MCS.

One of the important finding of our study, the low mean MCS score (39.8) associated with khat chewers in the end of the treatment compare to non khat chewers (47) (Table 6). This clearly reflects the risk of depression among khat chewers at the end of the treatment which may affect the outcome of TB treatment.likewise, Fig 3 compared mean MCS score for khat and non khat chewers and shows that khat chewers had lowest score at each point. Likewise, Study performed in Yemen to find the effectiveness of khat in mood by using Hospital anxiety and depression scale found that khat leads to mood disturbance due to sympathomimetic effect of cathinone on CNS [56]. Moreover, a Study performed in Yemen indicates that khat chewing result in health complication such as stress, sleep disturbances, and anorexia [19] [57] [58] [24], which may affect the QoL of patients and consequently result in poor treatment outcome.

**Fig 4:**
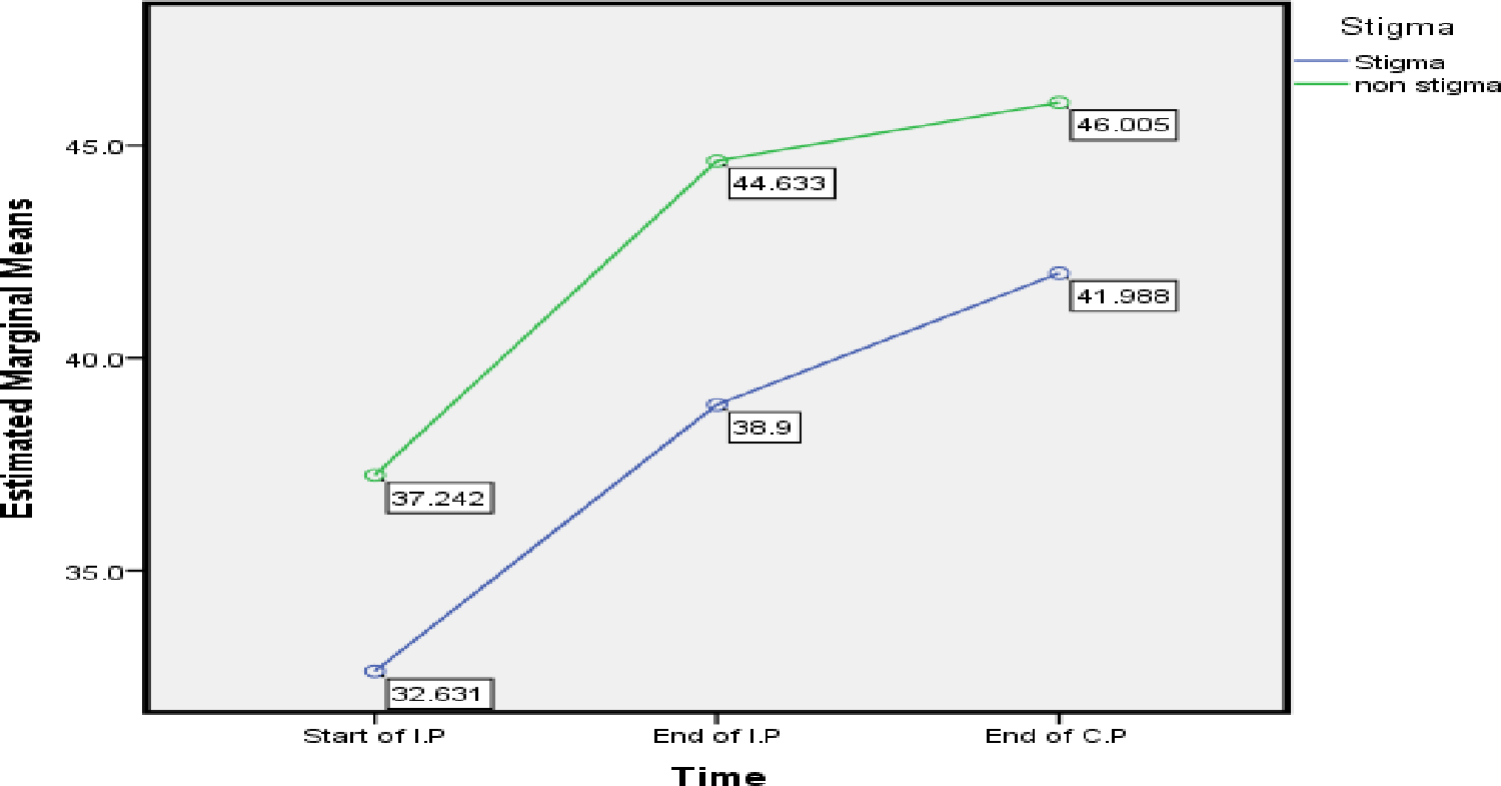
Stigma and Non Stigma Status: Difference in estimated marginal means of MCS.

With respect to stigmatization, Stigma can play a major role in affecting Qol of TB patient and may lead to poor quality of life [59], in addition, it can result in default or rejection of treatment due to isolation from surrounded society [60] [61]. Our finding shows a difference of mean MCS score between stigmatization and non-stigmatization patient (Table 6). Likewise, Fig 4 shows that a Stigmatization TB Patients got low MCS score at each point compares to those non stigma TB patient. This result was similar to study states that stigma associated with TB had terrible effect on emotional quality of TB patient [10] [59]. Contrary to our study, [62] conducted a study in the urban area of Delhi conclude that there is no effect of Stigma on QoL of TB patient. Moreover, [44] state that effect of stigma on HRQoL of TB patient in development countries is unknown.

**Fig 5:**
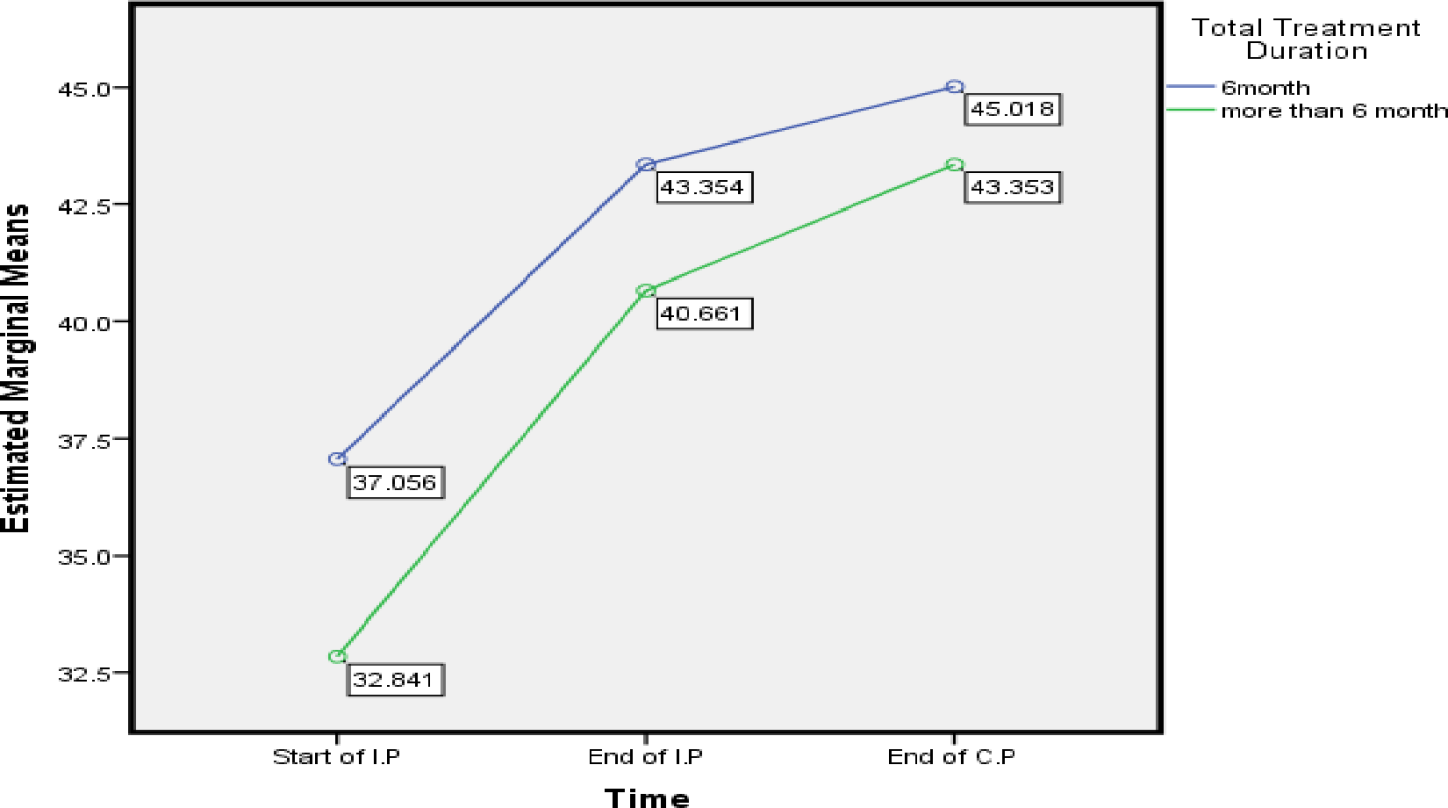
Total Treatment Duration: Difference in estimated marginal means of MCS.

With respect to duration of TB treatment, from Fig 5 it is clearly shown that TB patient toke medication more than 6 months got worse MCS at three time point compare to patient complete the treatment within 6 months. Treatment considers prolonging if the treatment duration exceeds 6 months [63]. Moreover, [25] state that only a few studies found the influence of duration of treatment on HRQoL, for example, [13] state that duration of treatment affect QoL significantly. Other Study performed to find the difference of QoL between active and inactive TB, found a positive significant relation between the duration of treatment and MCS score in active TB [53]. Contrary to our result [35] found that Patients who completed an 8 months course of TB therapy had significantly higher HRQoL scores. Moreover [53] report that only MCS score improved with increase of duration of treatment. Moreover, our finding shows that PCS mean score did not show any difference with respect to duration of treatment concluding that, duration of treatment more than 6 months can result in poor MCS score of TB patients which also may lead to poor treatment outcome due to depression.

### Recommendation

We recommend extensive care and support to TB patients in the first two months of treatment to improve HRQoL, particularly for MCS because, it can result in patient defaulters. In addition, other factors such as chewing khat habit, stigmatization, and duration of treatment more than 6 month should be considered during TB treatment as it may result in poor outcome due to the negative role of MCS score. Also, more effort should be focus by health workers in Alhodidah city to increase the MCS quality of life because of low educational level of this governance. The National TB Control Program should provide new TB equipment and facilities to other cities and to rural areas to improve the clinical outcome of TB patients.
